# A Search for Secondary Hypertension: “Where’s Waldo?”

**DOI:** 10.7759/cureus.15698

**Published:** 2021-06-16

**Authors:** Seda Tolu, Neelja Kumar, Shitij Arora

**Affiliations:** 1 Medicine, Montefiore Medical Center, Bronx, USA; 2 Nephrology, Montefiore Medical Center, Bronx, USA; 3 Internal Medicine, Montefiore Medical Center, Bronx, USA

**Keywords:** cardio vascular disease, outpatient management, hypertension, medical screening, secondary hypertension, general nephrology

## Abstract

Hypertension is an important modifiable risk factor for cardiovascular disease and stroke. Most cases are diagnosed as essential hypertension however, in some patients, a secondary (and potentially curable) cause is identified. Selecting the right patient to screen for a secondary cause can be challenging and certain clinical and laboratory characteristics can guide work-up. We report a case of a 67-year-old man who presented with intracranial hemorrhage. He had a history of resistant hypertension for three decades and chronic hypokalemia while on a non-diuretic antihypertensive regimen. We discuss our approach to a hypertensive hypokalemic phenotype that led to the diagnosis of Liddle’s syndrome with complete amelioration of hypokalemia with directed therapy. This case highlights the importance of accurate and early screening for causes of secondary hypertension in the outpatient community, and in doing so, preventing downstream catastrophic outcomes. It is imperative to develop a clear, concise approach to secondary hypertension, and raising awareness for the importance of early diagnosis as it can potentially avoid downstream sequela.

## Introduction

Hypertension is an important modifiable risk factor for cardiovascular disease and stroke. In about 10% of patients with hypertension, a secondary (and potentially curable) cause is identified. Secondary hypertension is defined as hypertensions that persist despite three or more blood pressure medications at appropriate doses, including one diuretic [1-3]. Selecting the right patient to screen for a secondary cause can be challenging and certain clinical and laboratory characteristics can guide work-up. Common causes of secondary hypertension in adults according to prevalence include obstructive sleep apnea, primary hyperaldosteronism, and reno-vascular hypertension [1]. This case report aims to provide a clear, concise approach to secondary hypertension, and raise awareness for the importance of early diagnosis as it can potentially avoid catastrophic downstream sequela.

## Case presentation

**Figure 1:**
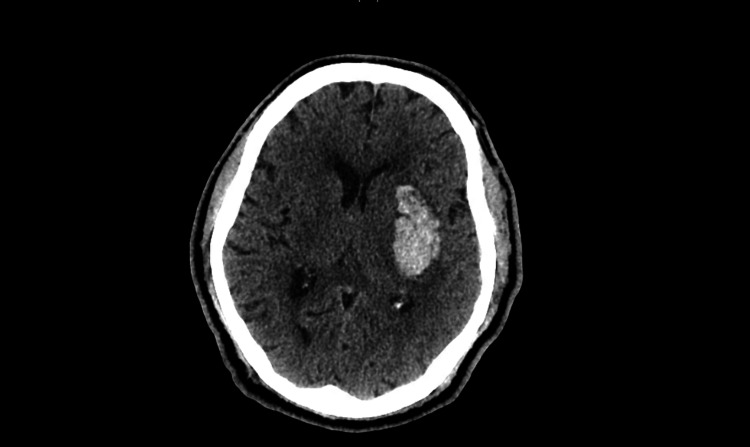
CT head without contrast showing acute left basal ganglia hematoma Acute left basal ganglia hematoma (volume approximately 16.5 mL) with local mass effect and partial effacement left lateral ventricle. Minimal left to right shift of midline at the level of the septum pellucidum.

A 67-year-old Italian male with over three decades of hypertension and chronic hypokalemia, treated with three non-diuretic antihypertensives, and 40-80meq of potassium chloride daily was admitted with sudden onset of altered mental status, right-sided hemiparesis, and dysarthria. Blood pressure upon arrival to the emergency department was 190/115 mmHg. Initial CT scan revealed an acute left basal ganglia hemorrhage requiring emergent craniotomy and hematoma evacuation (Figure 1).

**Figure 2:**
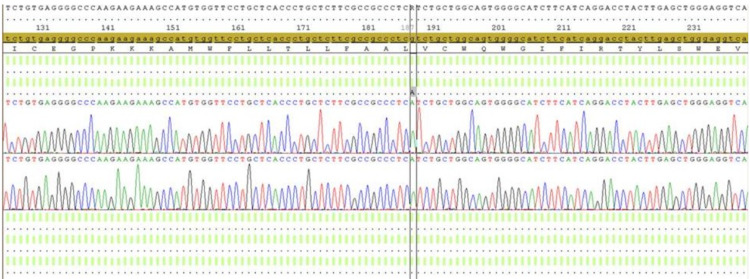
Chromatogram representation of genetic mutation in the SCNN1B gene Chromatogram displaying single point mutation with substitution of guanine to adenosine in position 187 of exon 2 in the SCNN1B gene.

Laboratory data was notable for persistent hypokalemia as low as 2.5 mEq/L (reference range: 3.5-5.0), metabolic alkalosis with bicarbonate levels ranging from 30-34 mEq/L (reference range: 22-29) and preserved renal function (creatinine 0.70 mg/dL, glomerular filtration rate [GFR] >120 mL/min/BSA). After ruling out transcellular shift and non-renal losses of potassium, we focused on renal losses, with further investigation of the aldosterone axis. Aldosterone level was 5 ng/dL in sitting position (reference range: 3-16) and renin was 0.13 ng/ml/h (reference range: 0.25-5.82). Morning cortisol was normal at 21.4 ug/dL (reference range: 5-25). Suspecting a low renin etiology of hypertension, gene sequencing for monogenic causes of hypertension was performed which identified a mis-sense mutation in the SCNN1B gene (p.Val63Ile), confirming the diagnosis of Liddle’s syndrome (Figure 2).

The patient was started on amiloride with robust rescue of potassium levels without the need for further potassium supplementation and normalization of blood pressures.

## Discussion

**Figure 3:**
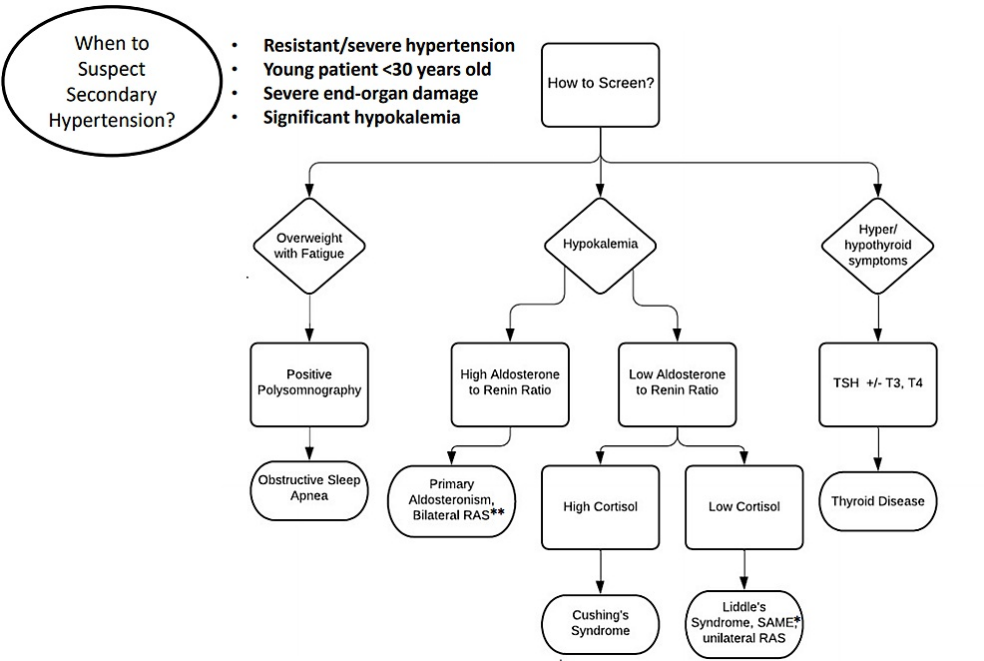
When to suspect and how to screen for secondary hypertension *Syndrome of Apparent Mineralocorticoid Excess, **Renal Artery Stenosis

Hypertension is an important modifiable risk factor for cardiovascular disease and stroke. In 10% of patients with hypertension, a secondary (and potentially curable) cause is identified [4]. Selecting the right patient to screen for a secondary cause can be difficult. However, having an easy-to-use approach for all adults with suspected secondary hypertension can lead to earlier diagnostic accuracy of underlying causes, allowing for directed treatment and prevention of end-organ damage. With this case, we discuss our approach for when to suspect and how to screen for secondary hypertension (Figure 3).

The three most common causes of secondary hypertension include obstructive sleep apnea, primary hyperaldosteronism, and reno-vascular hypertension [5]. Those under the age of 30, with resistant hypertension, defined as being on three anti-hypertensives of different classes, one of which is a diuretic, or with significant hypokalemia, should prompt workup for secondary hypertension.

Those with a hypokalemic hypertensive phenotype should be screened for hyper-aldosteronism which can be reliably tested for by the aldosterone-renin ratio (ARR). Mid-morning samples of plasma aldosterone and renin taken after a patient has been seated for 5-10 minutes and while on a diet liberal in salt before testing is considered to be ideal. Correction of hypokalemia and stopping aldosterone antagonists for 4-6 weeks prior to testing is recommended. An elevated ARR suggests hyper-aldosteronism as in aldosterone secreting adrenal adenoma (primary aldosteronism) or reno-vascular hypertension including renal fibromuscular dysplasia (secondary aldosteronism) [6].

Resistant hypertension with hypokalemia but with low renin-low aldosterone syndromes (low ARR), as in our case, is seen in monogenic disorders such as Syndrome of Apparent Mineralocortocoid Excess (SAME) and Liddle’s syndrome. SAME is a genetic disorder caused by an inactivating mutation in the 11-beta-hydroxysteroid dehydrogenase enzyme type 2 isoform (11-beta-HSD2). 11-beta-HSD2 normally converts cortisol to cortisone, latter has no mineralocorticoid activity. However, cortisol has an affinity to the mineralocorticoid receptor and behaves like aldosterone. An acquired SAME syndrome can also be seen with chronic licorice ingestion as it inhibits 11-beta-HSD2. The hypokalemic and low ARR phenotype of our patient promoted further workup for monogenic causes of hypertension and that revealed a mutation in the SCNN1B gene. This gene encodes a constitutively active ENaC leading to sodium retention, hypertension, and hypokalemic metabolic alkalosis as seen in Liddle’s Syndrome [7-8]. Though the prevalence of Liddle’s Syndrome is rare, his persistently hypokalemic phenotype and relatively early-onset hypertension (early 30s) warranted workup for secondary hypertension.

## Conclusions

In the famous Martin Handford books, ‘Waldo’ is a bespectacled cartoon character wearing a red-striped shirt and cap. The challenge for the reader is to identify him in this massive crowd as he is mingled with other similar striped red herrings. Multiple perceptual and cognitive processes come together to identify ‘Waldo’ in a cluttered scenario and there are times when despite being obviously in front the brain fails to recognize the image. Amongst the many patients with hypertension seen in practice, identifying a correctable cause of hypertension can be crucial in preventing catastrophic outcomes and end-organ damage.
